# Imaging diagnosis of intracranial atherosclerosis stenosis-related large vessel occlusion before and during endovascular therapy

**DOI:** 10.3389/fneur.2023.1168004

**Published:** 2023-06-21

**Authors:** Takeshi Yoshimoto

**Affiliations:** Department of Neurology, National Cerebral and Cardiovascular Center, Suita, Japan

**Keywords:** imaging, diagnosis, intracranial atherosclerosis stenosis, large vessel occlusion, endovascular treatment

## Abstract

It is becoming increasingly important to identify the type of stroke, especially the mechanism of occlusion, before and during its treatment. In the case of intracranial atherosclerotic stenosis-related large vessel occlusion, it is necessary to develop a treatment strategy that includes not only mechanical thrombectomy but also adjunctive therapies such as primary or rescue therapy (percutaneous angioplasty, intracranial/carotid stenting, local fibrinolysis) and perioperative antithrombotic therapy. However, in clinical practice we often encounter cases where it is difficult to identify the occlusive mechanism before endovascular treatment because of insufficient information in the minimal circumstances of the hyperacute phase of stroke. Here we focus on the imaging diagnosis before and during treatment of intracranial atherosclerotic stenosis-related large vessel occlusion with *in situ* thrombotic occlusion as the mechanism of thrombotic occlusion, based on previous reports. We describe the diagnosis of intracranial atherosclerotic stenosis-related large vessel occlusion from the perspectives of “thrombus imaging,” “perfusion,” and “occlusion margin.”

## 1. Introduction

Considering the extensively documented effectiveness and safety of mechanical thrombectomy (MT) in the management of acute ischemic stroke (AIS) due to intracranial large vessel occlusion (LVO) (1). It is increasingly crucial to prioritize the first-pass effect (2) and achieve successful endovascular treatment (EVT) without exacerbating intracranial hemorrhage, thus elevating the standard of care. The classification of stroke, particularly the occlusion mechanism, is becoming increasingly significant both preoperatively and during treatment. Embolic LVO is often the preferred indication for mechanical thrombectomy (MT) when an embolic source is discerned before the procedure. In cases of intracranial atherosclerotic stenosis (ICAS)-related LVO, it is imperative to design a treatment regimen that incorporates not only MT but also auxiliary therapies such as primary or rescue therapy (percutaneous angioplasty, intracranial/carotid stenting, and local fibrinolysis) and perioperative antithrombotic therapy. However, accurately determining the occlusion mechanism before EVT can prove challenging in clinical practice due to a dearth of information regarding the patient’s medical history, pre-existing conditions, and comorbidities in the hyperacute stage of stroke. A full understanding of the situation is often not achieved until after EVT. Moreover, intracranial atherosclerotic stenosis (ICAS)-related large vessel occlusion (LVO) is more commonly observed in East Asia, with a frequency of 15–25% (3, 4), as compared to Europe and the United States. Studies have demonstrated that ICAS-related LVO has a lower success rate of recanalization, a longer duration to successful recanalization, and worse outcomes as compared to embolic LVO (5, 6), underscoring the critical need to enhance the accuracy of etiologic diagnosis before initiating treatment.

This paper will focus on the imaging diagnosis of intracranial atherosclerotic stenosis (ICAS)-related large vessel occlusion (LVO) with *in situ* thrombotic occlusion as the occlusion mechanism, with reference to previous research. The diagnosis of ICAS-related LVO will be described from the perspectives of “thrombus imaging,” “perfusion imaging,” and “occlusion margin imaging.”

## 2. Thrombus imaging

Recent research has shown that thrombi associated with cardioembolism have a greater proportion of fibrin compared to red blood cells, whereas those associated with intracranial atherosclerotic stenosis (ICAS)-related large vessel occlusion (LVO) have a higher concentration of red blood cells. The type of occlusive thrombus is closely linked to the mechanism of occlusion (7, 8). Non-contrast computed tomography (CT) and magnetic resonance imaging (MRI) are imaging modalities capable of visualizing the occlusive thrombi. In this context, we will discuss the “hyperdense middle cerebral artery (MCA) sign” detected on Non-contrast CT (NCCT) and the “susceptibility vessel sign (SVS)” detected on MRI scans.

### 2.1. Hyperdense MCA sign

**Figure 1:**
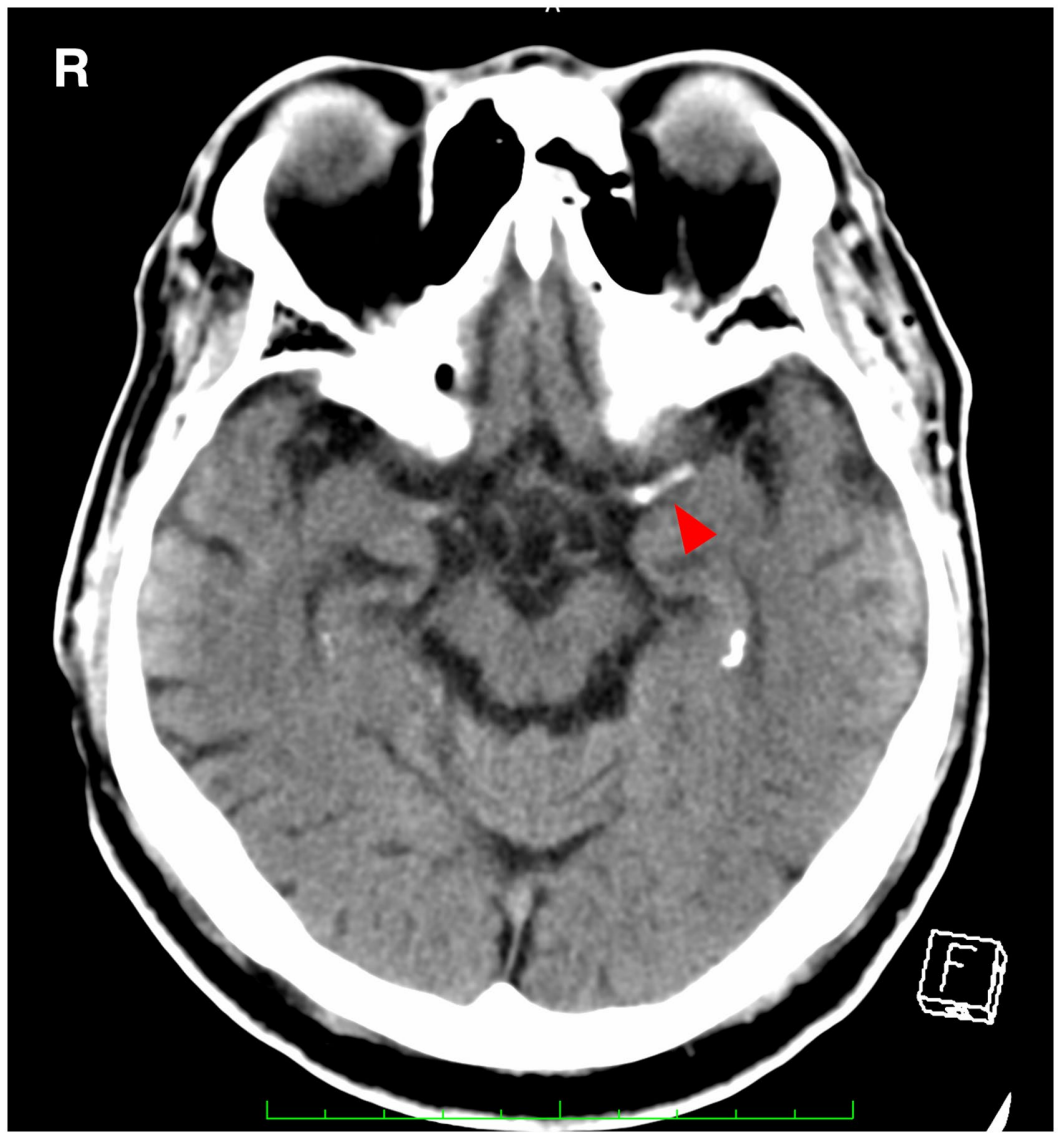
A hyperdense MCA sign (arrowhead) is seen in the left middle cerebral artery. MCA, middle cerebral artery.

The hyperdense middle cerebral artery (MCA) sign, as illustrated in Figure 1, has been observed to be associated with embolic large vessel occlusion (LVO) (9, 10). The appropriate threshold for detecting thrombus has been established as a Hounsfield Unit (HU) value of 51, which exceeds the standard value of 45 HU (11). Intracranial arterial wall calcification is a hallmark of atherosclerosis and has been correlated with lower rates of reperfusion following thrombectomy (12). A substudy within the MR CLEAN trial enrolled 344 patients, with 156 in the endovascular treatment (EVT) group and 188 in the control group, excluding individuals who were difficult to evaluate due to 3-mm ultraslice imaging or body motion. The results indicated significant differences in reperfusion rates and outcomes based on the type of intracranial carotid artery calcification. Patients with medial calcification, i.e., calcification within the occluded vessel, had better outcomes in the EVT group compared to the control group [adjusted common odds ratio (OR), 2.32; 95% confidence interval (CI), 1.23–4.39], while endovascular therapy did not have a significant impact on patients with intimal calcification (adjusted common OR, 0.82; 95% CI, 0.40–1.68) (12). In addition, a study on quantitative HU values measured the HU of intra-arterial radiation in 102 consecutive Chinese stroke patients who underwent multiphase CT angiography and EVT within 6 h of onset and examined the HU distal/proximal ratio to predict emboli The optimal cutoff was an HU ratio < 0.6 measured at 2 mm from the embolization site (area under the maximum curve = 0.87, sensitivity 96%, specificity 81%) (13).

### 2.2. Susceptibility vessel sign/2-layered susceptibility vessel sign

**Figure 2:**
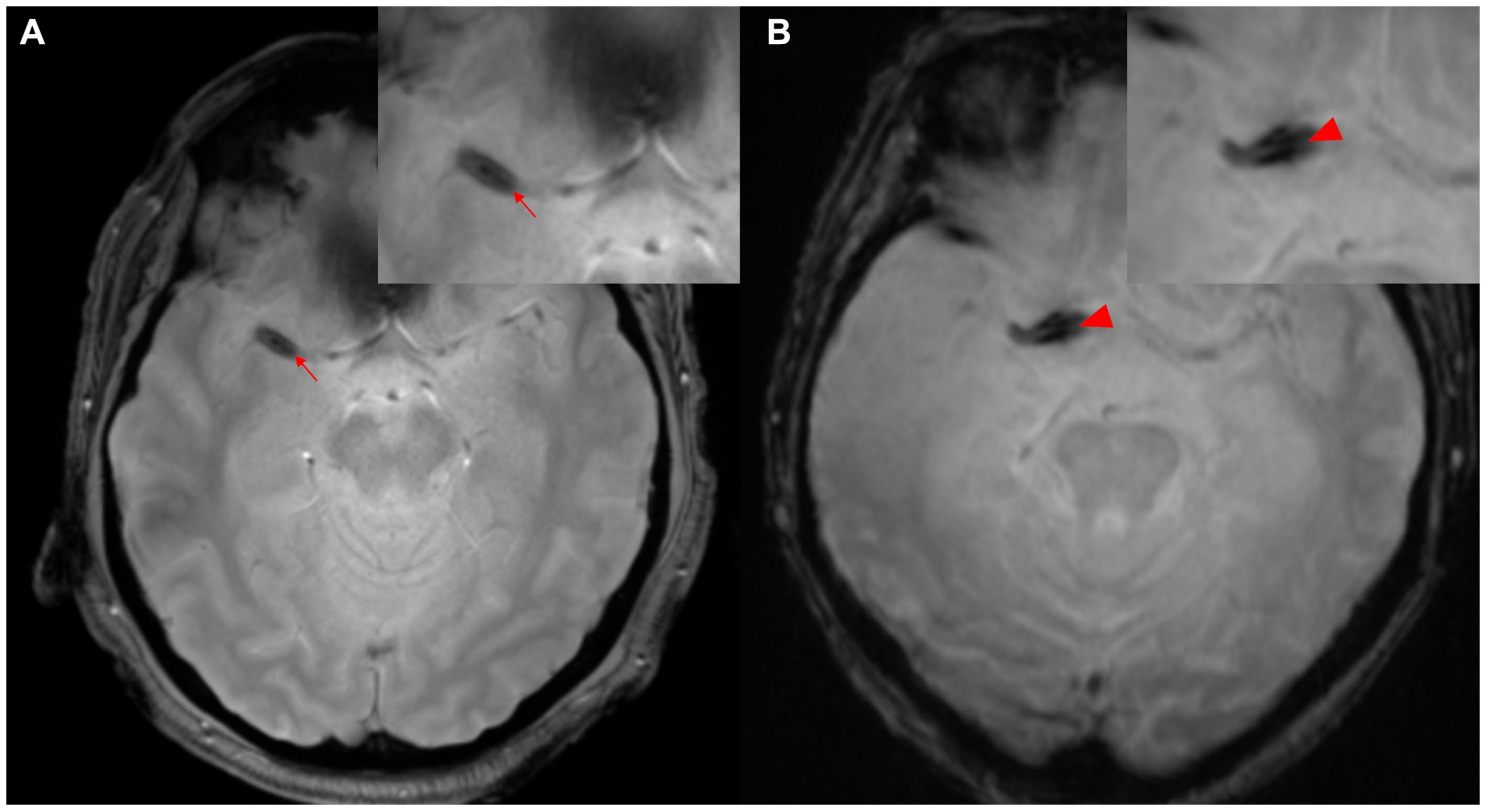
Two-layer SVS. **(A)** SVS (arrowhead) is seen distal to M1 of the right MCA. **(B)** SVS (arrowhead) is seen in the right internal carotid artery proximal to M1 segment of MCA. MCA, middle cerebral artery; SVS, susceptibility vessel sign.

The concept of SVS has been classically defined as “a manifestation of low signal intensity in occluded thrombi on T2*-weighted Gradient Echo (GRE) images, caused by the magnetic susceptibility effect of deoxyhemoglobin in red blood cells” (14). The diagnostic value of SVS in ischemic stroke is widely acknowledged, with a prevalence of 77.5% in cases of cardioembolism (15). This is particularly evident in thrombi with high concentrations of red blood cells (known as “red thrombi”) (16). Moreover, the presence of GRE-SVS has been associated with cardioembolism and spontaneous recanalization of occluded vessels (17, 18). The diameter of SVS has been independently linked to the likelihood of cardioembolism (adjusted odds ratio [OR], 1.97; 95% confidence interval [CI], 1.34–2.90; *p* < 0.01). However, it is important to note that a diagnosis of SVS does not necessarily indicate cardioembolism, as 25.5% of patients without cardioembolism also exhibit SVS (14). In cases of the middle cerebral artery (MCA) diagnosed *via* cerebral angiography, the sensitivity of SVS was higher than that of the hyperdense MCA sign NCCT (82% vs. 54%) (14). The concept of “2-layer SVS” (as shown in Figure 2) on 3-T MR T2*-weighted GRE has been reported in Japan, where 47.7% of 132 patients (72 men, mean age 74.5 years) were diagnosed with cardioembolism. The sensitivity of SVS for cardioembolism and large-artery atherosclerosis was not statistically significant (74.6% vs. 58.0%), but the sensitivity of 2-layer SVS alone was found to be significantly higher for cardioembolism (42.9%) than for large-artery atherosclerosis (2.9%; *p* < 0.001) (19). The specificity of 2-layer SVS for cardioembolism and the diagnostic ratios were 97.1 and 25.1%, respectively (42.0 and 2.1% for SVS). These findings may be attributed to the magnetic heterogeneity within the thrombus, suggesting a significant correlation between 2-layer SVS and higher thrombus weight and red blood cell components (19–21).

## 3. Perfusion imaging

A substantial distinction exists in the cerebral perfusion status preceding the manifestation of LVO AIS between ICAS-LVO and embolic LVO. The former is characterized by a chronic reduction in cerebral perfusion due to severe ICAS and *in situ* thrombus occlusion, while the latter is distinguished by the absence of abnormal intracranial perfusion and the acute reduction in perfusion caused by emboli. The diagnostic differentiation between the two forms of LVO AIS may be facilitated by evaluating the cerebral perfusion status through MR perfusion or CT perfusion.

### 3.1. Perfusion profile (*T*_max_, hypoperfusion intensity rate)

**Figure 3:**
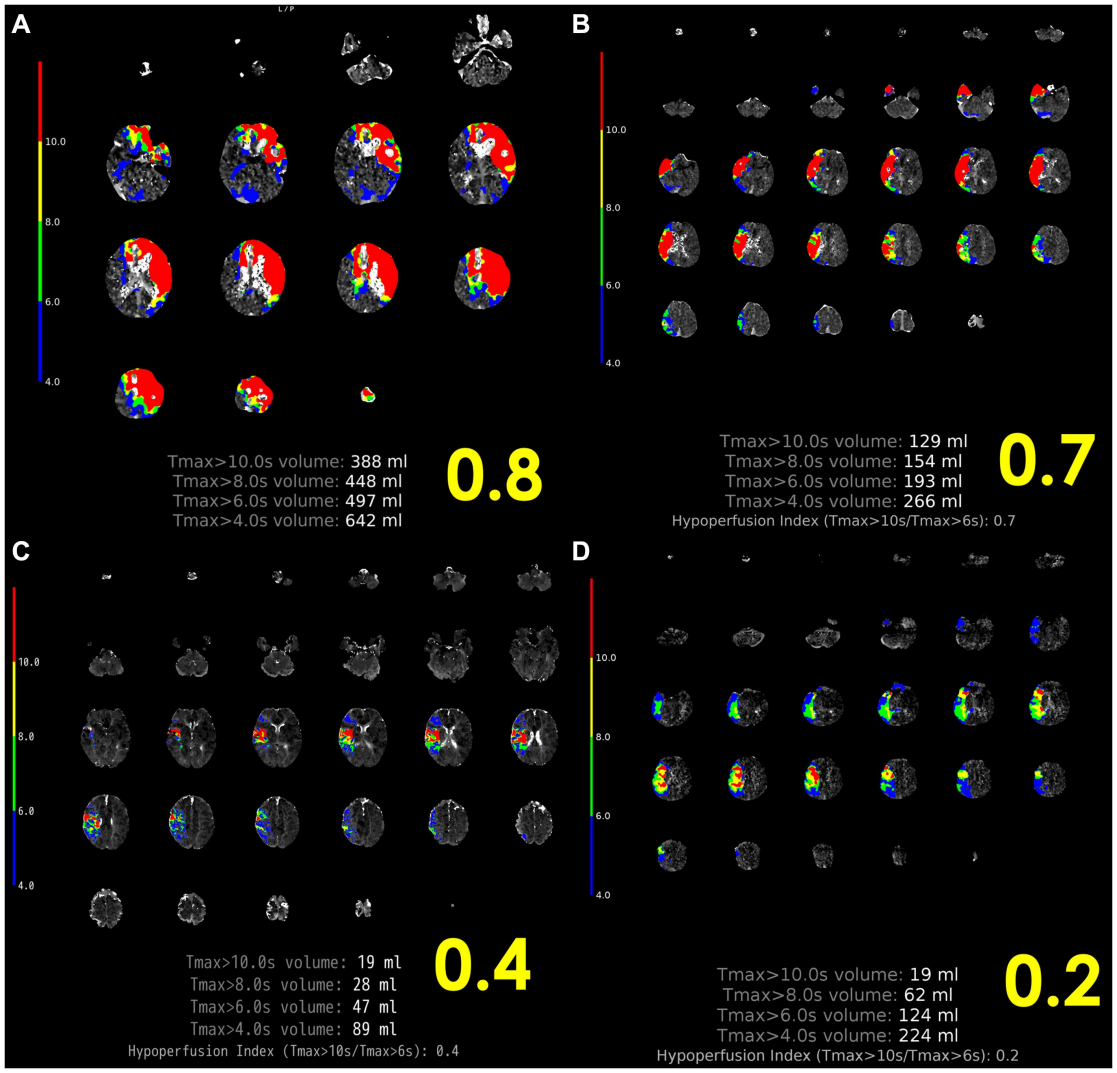
Representative case of HIR. **(A)** Left internal carotid artery occlusion, 2 h after onset, HIR 0.8, rapid progression, cardiogenic cerebral embolism. **(B)** Proximal occlusion of the right MCA M1, 10 h after last healthy control, HIR 0.7, rapid progression, other cerebral infarction (cerebral embolism with unknown embolic source). **(C)** Distal occlusion of right MCA M2, onset 4 h, HIR 0.4, immediate progression, cardioembolism. **(D)** Proximal occlusion of the right MCA M1, HIR 0.2, slow progression, ICAS-related LVO. ICAS-related LVO, intracranial atherosclerotic stenosis-related large vessel occlusion; HIR, hypoperfusion intensity ratio; MCA, middle cerebral artery; SVS, susceptibility vessel sign.

The EPITHET-DEFUSE study disclosed a correlation between cerebral perfusion imaging attributes and atrial fibrillation. Of the 124 patients who underwent perfusion imaging and were enrolled in DEFUSE (22) or EPITHET (23), 28 patients were designated as the “definite AF group,” having been identified as having atrial fibrillation (AF) upon admission, while the remaining 96 were classified as the “NO AF group.” The comparison of perfusion imaging profiles revealed that the definite AF group displayed elevated profiles relative to the NO AF group, as indicated by higher time to maximum concentration (*T*_max_) values (*T*_max_ > 4 s: 136 mL vs. 81 mL, *p* < 0.01; *T*_max_ > 6 s: 83 mL vs. 50 mL, *p* < 0.01; *T*_max_ > 8 s: 48 mL vs. 29 mL, *p* = 0.02) (24). The DEFUSE 2 (25) trial quantified hypoperfusion intensity rate (HIR) as *T*_max_ > 10 s divided by *T*_max_ > 6 s, with a median HIR value of 0.4 being linked to the extent of collateral bleeding (26). In a recent single-center observational study, HIR ≤0.22 (OR, 22.5; 95% CI, 2.9–177.0; *p* = 0.003) and cerebral blood volume index ≥0.9 (OR, 75.7; 95% CI, 5.8–994.0; *p* < 0.001) were found to be associated with ICAS-related LVO and to potentially predict underlying ICAS prior to EVT (27); (Figure 3). Moreover, cortical collateral vessels are also helpful in the preoperative differentiation of stroke etiology. Cortical vessels are prominent on prominent cortical vessels on susceptibility-weighted imaging (PCV-SWI) in 30.3% of ICAS-related LVO patients, whereas PCV-SWI is positive in only 13.4% of ICAS-related LVO patients, and PCV-SWI helps diagnose stroke etiology (28). Previous studies have also reported that prominent cortical and/or medullary veins on SWI can indicate to neuro-interventionists that the cause of LVO is more likely cardioembolism rather than ICAS-related LVO (29). A summary of previous reports of useful PWI diagnostic markers in the diagnosis of ICAS is shown in Table 1.

**Table 1:** Summary of previous reports of useful perfusion imaging diagnostic markers in the diagnosis of ICAS.

Year	Study design	Number	Patients	Diagnostic pathophysiology	Diagnostic markers
2015	Meta-analysis (EPITHET-DEFUSE) (24)	175	AIS, NIHSS >4 in EPITHET and > 5 in DEFUSE	No AF	Tmax profile (>8, >6 s, >4 s) volume
2018	Observational study (30)	250	Anterior Circulation LVO AIS	ICAS-LVO	Tmax >4 s/Tmax >6 s ratio ≥ 2
2021	Observational study (31)	42	symptomatic ICAS cases	Infarct pattern; internal borderzone	Δ Tmax >4 s – Tmax >6 s
2022	Observational study (32)	143	Anterior Circulation LVO AIS	LAA	HIR <0.4
2022	Observational study (27)	47	LVO AIS	LVO underlying ICAS	HIR ≤ 0.22 CBV index ≥0.9

AF indicates atrial fibrillation; AIS, acute ischemic stroke; CBV, cerebral blood volume; EVT, endovascular therapy; HIR, hypoperfusion intensity ratio; ICAS, intracranial atherosclerotic stenosis; LVO, large vessel occlusion; NIHSS, National Institutes of Health Stroke Scale; Tmax, time to maximum concentration.

## 4. Occlusion margin

In recent years various studies have documented the correlation between the characteristics of the proximal occluded vessel margin and LVO related to intracranial arteriosclerosis by utilizing initial cerebral angiography, which may prove valuable in the diagnosis of LVO related to intracranial arteriosclerosis prior to therapeutic intervention. In this context, here we focus on the features of the occluded vessel margin and recent relevant literature.

### 4.1. Significant fixed focal stenosis after reperfusion

**Figure 4:**
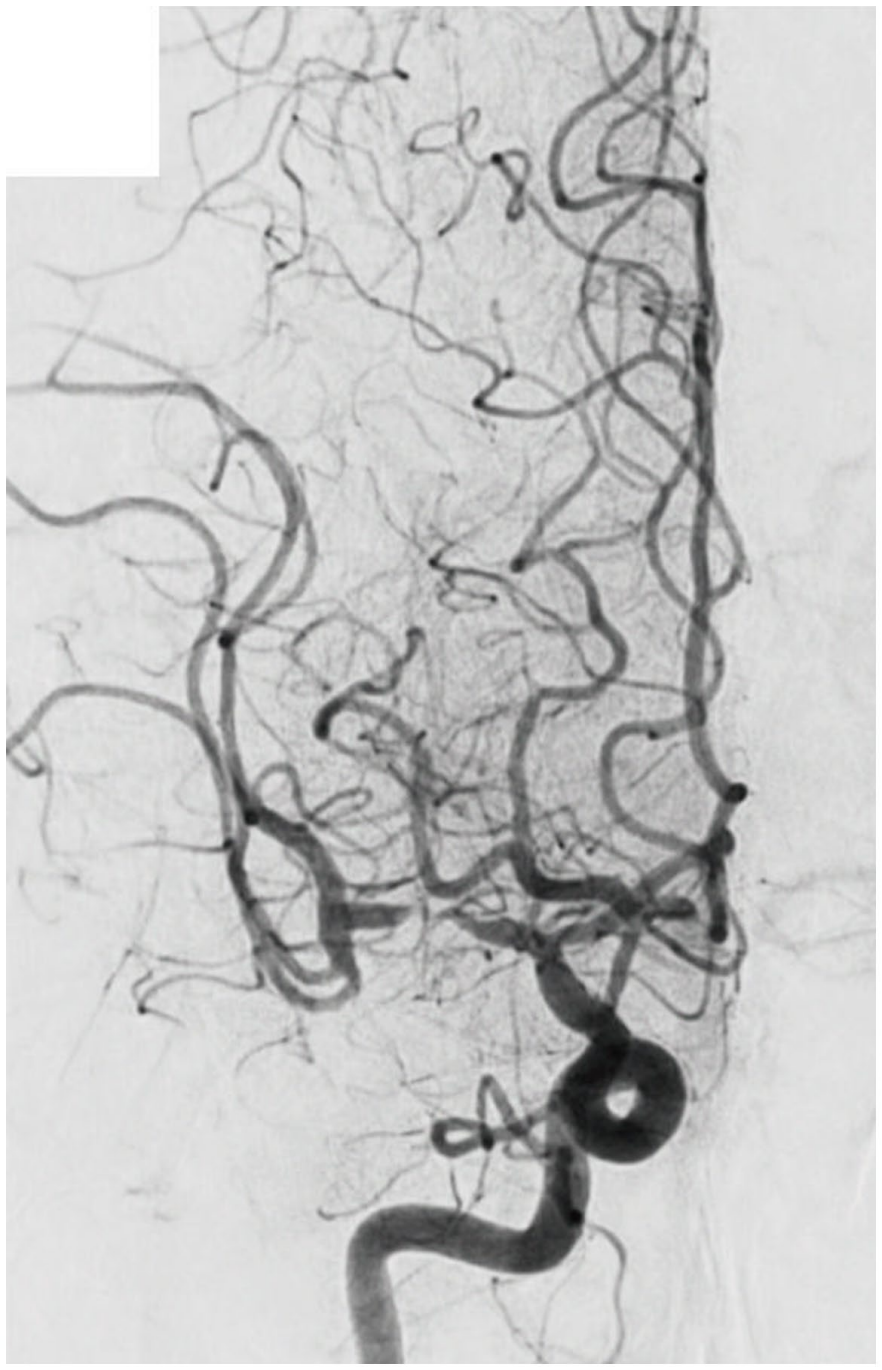
Significant fixed focal stenosis. A 59-year-old man was diagnosed with significant fixed focal stenosis. The right MCA M1 occlusion was recanalized in a single pass with a stent retriever, but the site of occlusion was found to have a fixed focal stenosis. MCA, middle cerebral artery.

“Fixed focal stenosis of substantial magnitude” has been proffered as a diagnostic hallmark of ICAS-related LVO (Figure 4) (34). This criterion is articulated as “a concentration of significant stenosis circumscribed to the site of occlusion,” as observed on postoperative or definitive imaging of the MCA and was initially a term employed to characterize ICAS-related LVO. Although few systematic investigations have focused on fixed focal stenosis, a Korean registry utilizing stent retrievers noted an incidence of approximately 15–20% (35).

### 4.2. Branching-site and truncal-type occlusion

**Figure 5:**
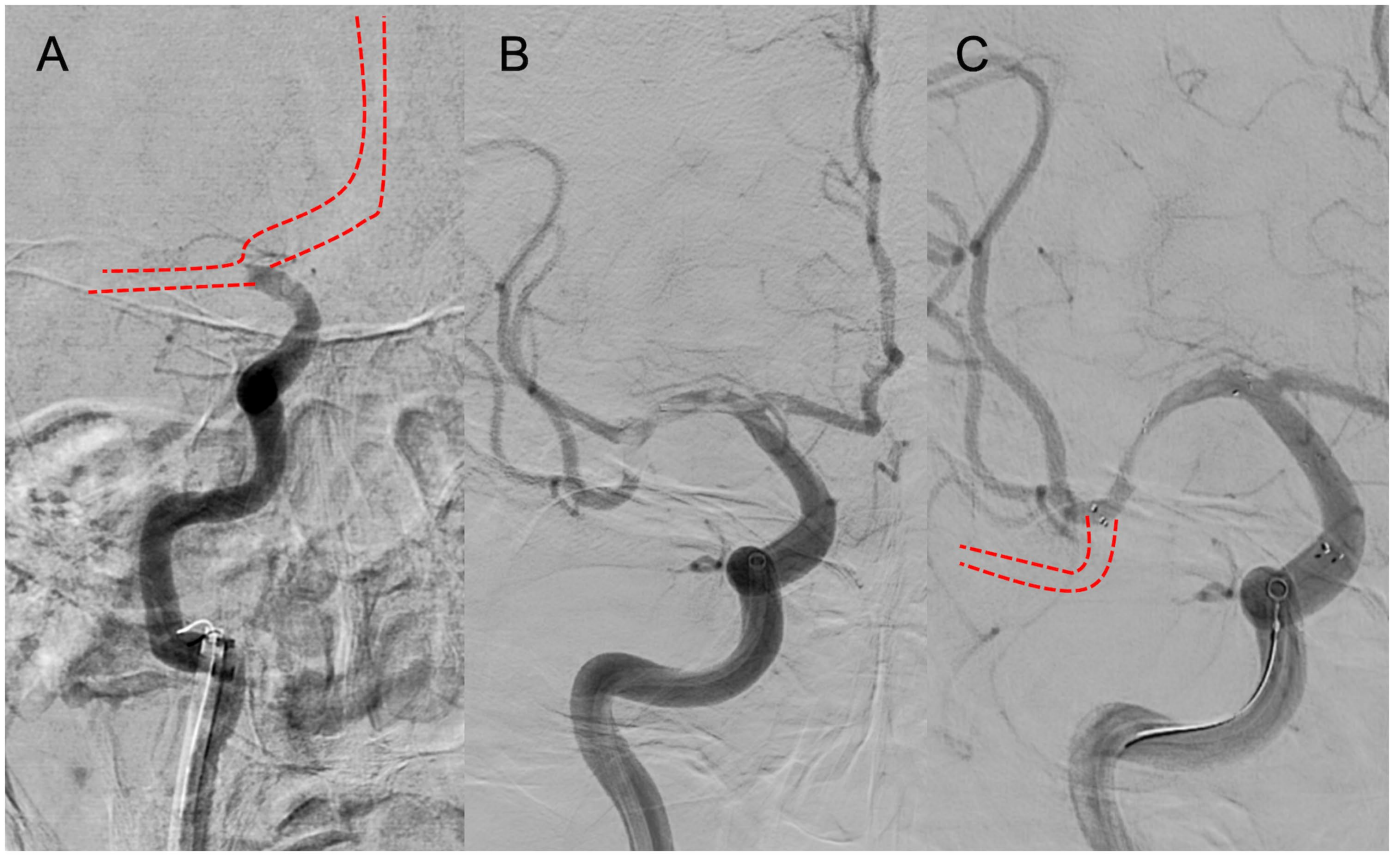
Branching-site occlusion. **(A)** Intracranial carotid artery occlusion without visualization of the anterior communicating artery (IC-T occlusion). **(B)** Y- or T-shaped visualization defect including vessel branches. **(C)** Partial or complete lack of visualization of vessel branches on angiography after stent retriever deployment.

**Figure 6:**
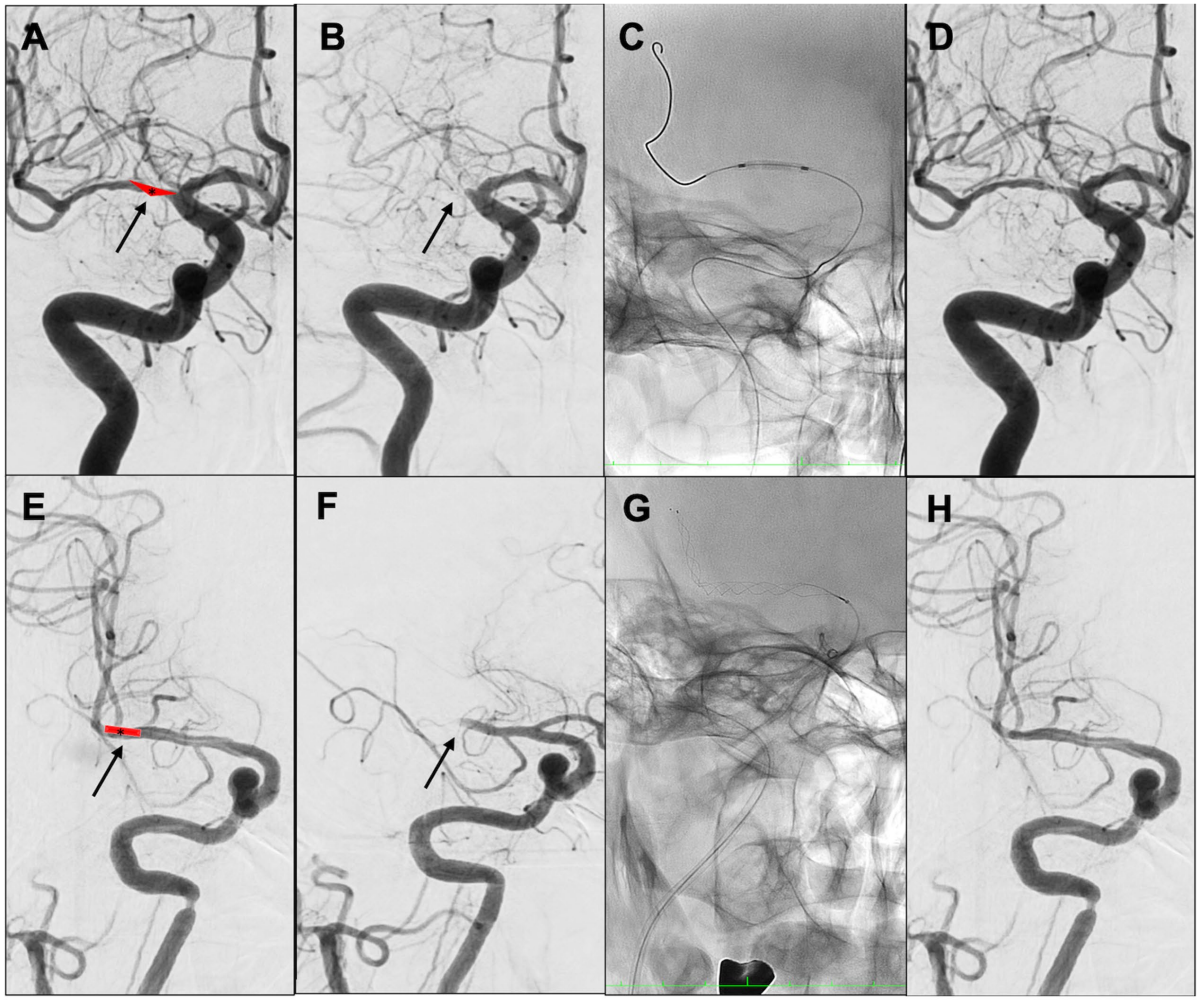
Representative cases of truncal-type occlusion/branching-site occlusion. **(A–D)** Truncal-type occlusion. **(E–H)** Branching-site occlusion. **(A)** Schema of *in situ* thrombus of right middle cerebral artery (MCA) M1 truncal type (asterisk). **(B)** Initial angiography shows occlusion of right MCA M1 (arrow). **(C)** Angioplasty. **(D)** Identification of residual stenosis in the right MCA M1 (ICAS-related LVO). **(E)** Embolic schema of the right MCA M1/M2 bifurcation (asterisk). **(F)** Initial angiography showed occlusion of right MCA M1 (arrow). **(G)** Stent retriever was deployed. **(H)** After retrieval of red thrombus, the bifurcation was found to be occluded. Angioplasty and stent retrieval were then performed.

A multicenter observational study from Korea determined that occlusions of a truncal type were correlated with a lack of responsiveness to stent retrievers and were the fundamental cause of strokes (36). The subjects were patients undergoing MT for intracranial occlusions of the internal carotid artery, MCA, proximal MCA, intracranial vertebral artery, or basilar cerebral artery. The occlusions were classified as either branching-site occlusion or truncal-type occlusion. Branching-site occlusion was defined as at least one of the following three conditions (Figure 5); (1) anterior communicating artery collateral flow that could not proceed to the contralateral ICA or MCA because it involved the internal cerebral artery bifurcation site (T occlusion); (2) direct visualization of a Y-or T-shaped filling defect involving a bifurcation site (Y-or T-shaped clot); and (3) another branch could not be visualized or was only partially visualized when the retriever was deployed to a branch across the occlusion site. Truncal-type occlusion, on the other hand, was defined as all branches and bifurcations visible beyond the occluded vessel, including those observed at recanalization. After a comprehensive evaluation involving chest electrocardiogram, echocardiography, cardiac CT, and cervical vascular echocardiography, the patients were classified as having embolic or non-embolic LVO. Of the 259 patients (mean age 70.3 years; male/female ratio 132:127), 83.4% had embolic LVO. Multivariate analysis revealed that younger age, prior coronary artery disease, and truncal-type occlusion were independently linked to the absence of embolic LVO (OR, 9.07; 95% CI, 3.74–22.0). Furthermore, truncal-type occlusion was associated with a higher frequency of reocclusion and a longer time to recanalization during stent retriever treatment. In a subanalysis of this study, truncal-type occlusion was associated with 93% of ICAS-related LVO and 10% of embolic LVO (*p* < 0.01) (35), whereas branching-site occlusion was associated with 7% of ICAS-related LVO and 90% of embolic LVO. In a separate study among 115 LVO patients in China, truncal-type occlusion was present in 93% of ICAS-related LVO and 10% of embolic LVO, while branching-site occlusion was observed in 7% of ICAS-related LVO and 90% of embolic LVO, yielding a significant difference between the two LVO types (*p* < 0.01 for each). The area under the curve of ICAS-related LVO in truncal-type occlusion was 0.916, with the sensitivity of 92.86% and specificity of 90.41% (36). CT angiography can also assess truncal-type occlusion, and although it is not a direct predictor of pathogenesis, branching-site occlusion as determined by CT angiography has been reported to independently predict the success of recanalization with stent retrievers (OR, 8.20; 95% CI, 3.45–19.5) (37). Representative cases of truncal-type occlusion/branching-site occlusion are shown in Figure 6.

### 4.3. Jet-like appearance

**Figure 7:**
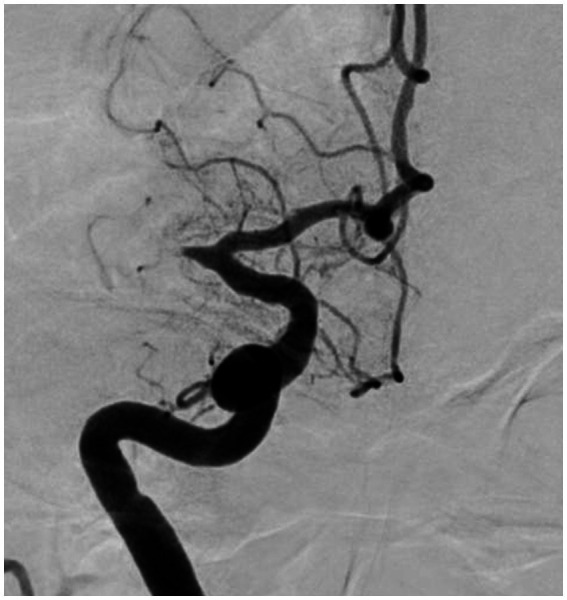
Jet-like appearance.

Jet-like appearance on cerebral angiography is characterized by a tapered end of the occluded vessel (Figure 7). In a Chinese observational study of 164 cases of LVO, 20.7% presented with this distinctive trait. Patients with the jet-like appearance were determined to be younger (mean age 68 years compared with 62.7 years) and had fewer severe symptoms (as indicated by a lower National Institutes of Health Stroke Scale [NIHSS] score of 16.6 compared with 12.4) than those without this feature. Multivariate logistic regression analysis revealed that a jet-like appearance was independently correlated with ICAS-related LVO (OR, 180.813; 95% CI, 17.966–1819.733; *p* < 0.001). The diagnostic performance of the jet-like appearance for identifying ICAS-related LVO was determined to have the sensitivity of 96%, specificity of 78%, and accuracy of 83% (38).

### 4.4. “Tapered” or “non-tapered”

**Figure 8:**
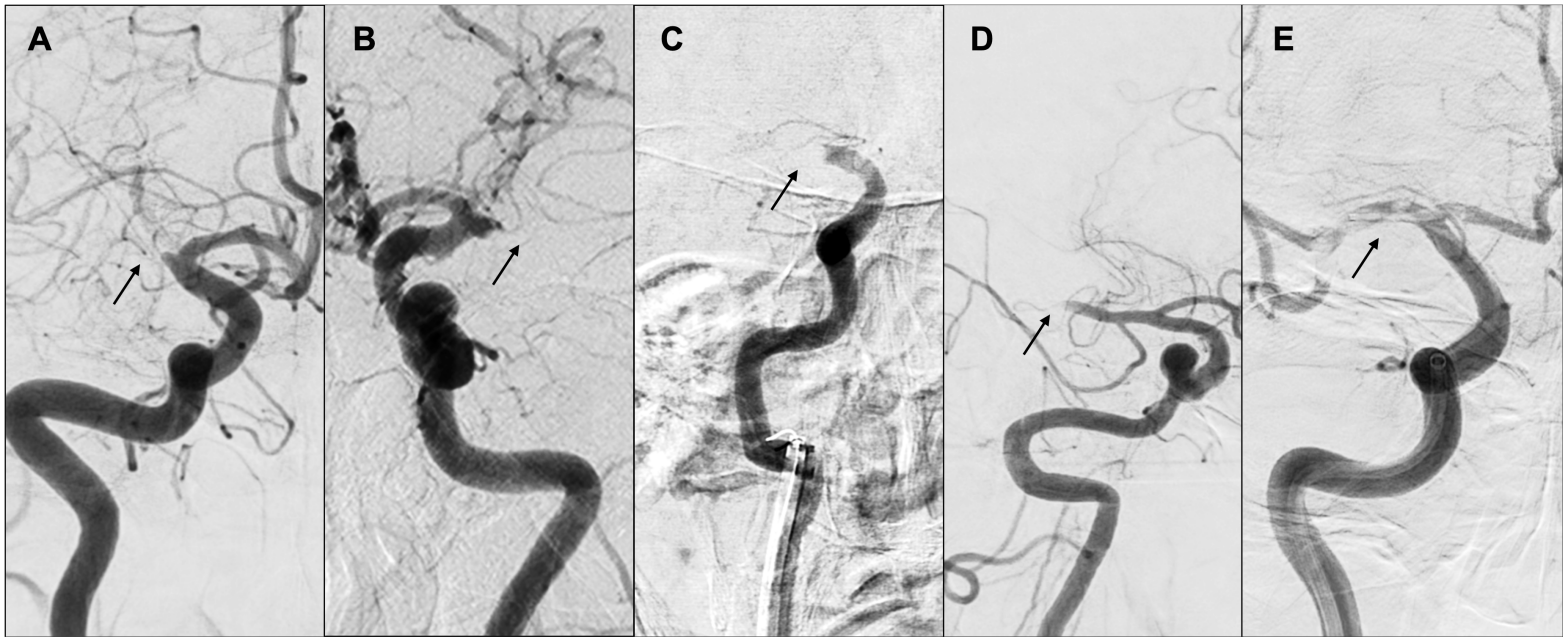
“Tapered” or “non-tapered” type. **(A,B)** Tapered; **(C)** meniscus; **(D)** cutoff; **(E)** tramtrack. Arrows indicate occlusion sites. **(A)** Right internal carotid arteriography showed the right middle cerebral artery (MCA) proximal to M1. The lumen of the occlusion gradually narrowed, and the occlusion site was severed at an acute angle at the superior wall of the artery. **(B)** Left internal carotid arteriography showed an acute occlusion angle in the distal left MCA M1, with the same pattern as in **(A)**. **(C)** Right internal carotid arteriography showed occlusion of the right internal carotid artery, concavity into the lumen representing meniscus occlusion. **(D)** Right internal carotid angiography showed distal M1 cerebral artery and cutoff occlusion. **(E)** Right internal carotid angiography showed partial occlusion of the right MCA from M1 proximal to M2, and multiple thrombus transillumination images indicating tramtrack occlusion were observed.

A Canadian study delineated two patient groups based on the occlusion location at the initial angiography and compared their demographic characteristics. Of 131 participants, 31 (23.6%) were classified as having a tapered presentation, while 100 (76.3%) were in the non-tapered group (Figure 8). The tapered group delivered a lower NIHSS score (10 vs. 16, with a significance level of *p* < 0.001), higher Alberta Stroke Program Early CT Score (9 vs. 7, with a significance level of *p* = 0.003), higher immediate reocclusion rate (26.7% vs. 8.2%, with a significance level of *p* = 0.025), and a lower rate of complete recanalization (45.2% vs. 71.0%, with a significance level of *p* = 0.028). The tapered group was also more likely to have LVO associated with ICAS (54.8% vs. 18.0%, with a significance level of *p* < 0.001) and to present with truncal-type occlusions (76.9% vs. 31.1%, with a significance level of *p* < 0.001) (39).

The benefits of identifying such occlusion margins through digital subtraction angiography are considerable, as they remain unaltered by therapeutic intervention and are thus useful in determining the causative mechanism of occlusion, even if complete recanalization of the affected vessel is not realized. Representative cases of “tapered” or “non-tapered” types are shown in Figure 8.

## 5. Conclusion

The diagnosis of ICAS-related LVO is informed by three key elements: “thrombus imaging,” “perfusion,” and “occlusion margin.” Preoperative assessment of the occlusion mechanism considers not only imaging results but also a comprehensive examination of these three elements along with factors indicative of ICAS-related LVO, such as progressive symptoms, low NIHSS scores, male sex, history of hypercholesterolemia and smoking, absence of AF, and posterior circulation strokes (33, 40, 41). It is imperative that a diagnosis is based on a holistic evaluation of these three elements as well as factors associated with ICAS-related LVO, including the absence of evidence of embolic LVO and posterior circulation stroke. In the case of LVO related to coronary artery disease, a non-embolic type of LVO, key patient histories characteristics such as young age and headache onset often play a significant role in clinical practice. There are limited systematic reports on the characteristic imaging findings before therapeutic intervention. Furthermore, there exist conditions, beyond ICAS-related LVOs, that warrant further investigation, such as unmet needs.

## Author contributions

The author confirms being the sole contributor of this work and has approved it for publication.

## Conflict of interest

The author declares that the research was conducted in the absence of any commercial or financial relationships that could be construed as a potential conflict of interest.

## Publisher’s note

All claims expressed in this article are solely those of the authors and do not necessarily represent those of their affiliated organizations, or those of the publisher, the editors and the reviewers. Any product that may be evaluated in this article, or claim that may be made by its manufacturer, is not guaranteed or endorsed by the publisher.
